# Characterization of common carp (*Cyprinus carpio* L.) interferon regulatory factor 5 (IRF5) and its expression in response to viral and bacterial challenges

**DOI:** 10.1186/s12917-016-0750-4

**Published:** 2016-06-27

**Authors:** Yaoyao Zhu, Chenchen Qi, Shijuan Shan, Fumiao Zhang, Hua Li, Liguo An, Guiwen Yang

**Affiliations:** Shandong Provincial Key Laboratory of Animal Resistance Biology, College of Life Science, Shandong Normal University, No. 88 East Wenhua Road, Jinan, 250014 People’s Republic of China

**Keywords:** IRF5, Common carp (*Cyprinus carpio* L.), Poly I:C, LPS, Gene expression

## Abstract

**Background:**

Common carp (*Cyprinus carpio* L.), one of the most economically valuable commercial farming fish species in China, is often infected by a variety of viruses. As the first line of defence against microbial pathogens, the innate immune system plays a crucial role in teleost fish, which are lower vertebrates. Interferon (IFN) regulatory factor 5 (IRF5) is a key molecule in antiviral immunity that regulating the expression of IFN and other pro-inflammatory cytokines. It is necessary to gain more insight into the common carp IFN system and the function of fish IRF5 in the antiviral and antibacterial response.

**Results:**

In the present study, we characterized the cDNA and genomic sequence of the IRF5 gene in common carp, and analysed tissue distribution and expression profile of this gene in response to polyinosinic:polycytidylic acid (poly I:C) and lipopolysaccharides (LPS) treatment. The common carp IRF5 (ccIRF5) gene is 5790 bp in length and is composed of 9 exons and 8 introns. The open reading frame (ORF) of ccIRF5 is 1554 bp, and encodes 517 amino acid protein. The putative ccIRF5 protein shares identity (65.4–90.0 %) with other fish IRF5s and contains a DNA binding domain (DBD), a middle region (MR), an IRF-associated domain (IAD), a virus activated domain (VAD) and two nuclear localization signals (NLSs) similar to those found in vertebrate IRF5. Phylogenetic analysis clustered ccIRF5 into the IRF5 subfamily with other vertebrate IRF5 and IRF6 genes. Real-time PCR analysis revealed that ccIRF5 mRNA was expressed in all examined tissues of healthy carps, with high levels observed in the gills and the brain. After poly I:C challenge, expression levels of ccIRF5, tumour-necrosis factor α (ccTNFα) and two IFN stimulated genes [ISGs (ccISG5 and ccPKR)] were up-regulated in seven immune-related tissues (liver, spleen, head kidney, foregut, hindgut, skin and gills). Furthermore, all four genes were up-regulated *in vitro* upon poly I:C and LPS challenges.

**Conclusions:**

Our findings suggest that IRF5 might play an important role in regulating the antiviral and antibacterial response in fish. These results could provide a clue for preventing common carp infection by pathogenic microorganisms present in the aquatic environment.

**Electronic supplementary material:**

The online version of this article (doi:10.1186/s12917-016-0750-4) contains supplementary material, which is available to authorized users.

## Background

Interferon (IFN) regulatory factors (IRFs) are key transcriptional mediators of virus-, bacteria- and IFN-induced signalling pathways, and they play an important role in antiviral defence, immune response, cell growth regulation, apoptosis and oncogenesis [1–3]. IRFs were originally identified as participating in the transcriptional regulators of IFN and IFN stimulated genes (ISGs) [4]. To date, eleven IRF family members have been identified in vertebrate, including IRF1, IRF2, IRF3, IRF4 [also known as PU.1 interaction partner (PIP), lymphoid-specific IRF (LSIRF) or consensus sequence-binding protein in adult T-cell leukemia cell line or activated T cells (ICSAT)], IRF5, IRF6, IRF7, IRF8 [also known as IFN consensus sequence binding protein (ICSBP)], IRF9 [also called as ISG factor 3 gamma (ISGF3γ)], IRF10 and IRF11. However, IRF10 and 11 have been identified in fish but in mammalians [5]. IRFs used to be classified into three groups according to differences in the C-terminal region: activators (IRF1, 3, 5, 7, 9 and 10), repressors (IRF2 and 8) and multifunctional factors that both activate and repress gene transcription (IRF2, 4, 5 and 7) [3, 4]. All members share a well-conserved N-terminal DNA-binding domain (DBD), and the conserved tryptophan repeat cluster in the first 120 amino acids of the DBD is responsible for binding the promoters of target genes [6]. IRF-associated domain (IAD) in the C-terminal of IRF3-11 mediates the interactions between IRFs and other protein to form transcriptional complexes [7].

Human IRF5 plays a crucial role in regulating the expression of IFN-α and IFN-β, mediating the virus- and cell type-specific immune response. IRF5 is also critical for the retinoic acid-inducible gene I (RIG-I) and Toll-like receptors immune pathways [8–12]. Other reports have also indicated that IRF5 may promote lymphocyte differentiation and apoptosis [9]. IRF5 knockout mice were reported to be susceptible to viral infections, while, the expression levels of type I IFNs and other pro-inflammatory cytokines including tumour-necrosis factor (TNF)-α, interleukin (IL)-6 and IL-12, were reduced in response to viral infections [12, 13].

IRF5 have been previously identified in several fish species, including grass carp (*Ctenopharyngodon idella*) [14], zebrafish (*Danio rerio*) [15], Atlantic salmon (*Salmo salar*) [16], Japanese flounder (*Paralichthys olivaceus*) [17], rock bream (*Oplegnathus fasciatus*) [18], half-smooth tongue sole (*Cynoglossus semilaevis*) [19], channel catfish (*Ictalurus Punctatus*) [20], Mi-iuy croaker (*Miichthys miiuy*) (unpublished data) and paddlefish (*Polyodon spathula*) [21]. Common carp (*Cyprinus carpio* L.), one of the most economically valuable commercial farming fish species, is often infected by a wide variety of viruses [22, 23]. To date, among the IRFs, only IRF3 and IRF7 have been identified in common carp [22]. Additional studies are needed to gain a better insight into the IFN system in common carp and the function of fish IRF5 in the antiviral and antibacterial immune response. In the present study, we characterized the cDNA sequence and genomic structure of IRF5 from common carp. We reported the evolutionary relationship of common carp IRF5 (ccIRF5) gene with other IRF genes. What’s more, we analysed its tissue-specific distribution in twelve tissues and expression profiles upon polyinosinic:polycytidylic acid (poly I:C) and lipopolysaccharides (LPS) stimulation both in vivo and in vitro by using real-time PCR.

## Methods

### Fish and poly I:C challenge

Common carp (body weight approximately 200 g) were obtained from the Fresh Water Fishery Research Institute of Shandong Province, China. Fish were reared in aerated freshwater tanks at 24–26 °C and were fed twice per day with commercial carp pellets (Shandong Tianshen Fishing Feedstuff Co., Ltd). After acclimatization for 1 week, the fish were used for the experiments.

Poly I:C (Sigma, USA) was re-suspended in phosphate-buffered saline (PBS) for the immune challenge experiments. Fish were intraperitoneally injected with 500 μl of a poly I:C solution (2.6 mg/ml) per fish, while un-challenged fish served as controls (indicated as 0 h). After injection, all fish were placed into a rectangular tank containing fresh water. At 0, 3, 6, 12, 24, 48 and 72 h post-injection (hpi), three fish in each group were euthanized and seven tissues including liver, spleen, head kidney, skin, gills, foregut and hindgut were sampled for total RNA extraction.

### RNA and genomic DNA extraction

Total RNA was extracted from different tissues of common carp using an RNA simple Total RNA Kit (TIANGEN) according to the manufacturer’s instructions. Total RNA quality and concentration were determined by 1 % gel electrophoresis and ultraviolet spectrophotometry, respectively. RNA samples had an OD260:OD280 ratio between 1.8 and 2.0. Genomic DNA was then removed and first-strand cDNA was synthesized by using the FastQuant RT Kit (containing gDNase) (TIANGEN) following the manufacturer’s protocol. Genomic DNA was extracted from the spleen of healthy fish using a TIANamp Genomic DNA Kit (TIANGEN).

### Cloning of cDNA and genomic sequences

A pair of primers, IRF5-F and IRF5-R (shown in Table 1), designed based on the conserved regions of different IRF5 genes, was used to amplify the corresponding ccIRF5 sequence from spleen cDNA of healthy fish. The PCR cycling parameters were 94 °C for 5 min, followed by 30 cycles of 94 °C for 30 s, 54 °C for 30 s, 72 °C for 50 s, and a final extension step of 72 °C for 10 min. Then, the full-length cDNA of ccIRF5 was obtained by the rapid amplification of the cDNA ends (RACE) method using a 3’-Full RACE and 5’-Full RACE Core Set Kit (TaKaRa). The detailed procedure was performed as described in the user manual, and two primers pairs (IRF5-3 F1/-3 F2 and IRF5-5R1/5R2) were used in the RACE-PCR (shown in Table 1).

**Table 1:** Primers used in this study

Primer	Sequence (5′-3′)	Application	GenBank No.
IRF5-F IRF5-R	GCAAGTACCAGGAAGGAG AAATCAGTTAAGGGCAACA	Homologous amplification	
IRF5-3 F1 IRF5-3 F2 IRF5-5R1 IRF5-5R2 IRF5g1-F IRF5g1-R	GTCACTCATTCTGTAGGCCCT GCAGAGAACCAGGTTCATCC TGTCCTTCGTGCCATCGTAGTG GTTTAGGGCACAGCGGAGATT CAGCACCATGAGTGGTCAACCACG AATATAGAGTTCTCCTCCTCCAG	First round 3′-RACE PCR Nested 3′-RACE PCR First round 5′-RACE PCR Nested 5′-RACE PCR For the first intron	
IRF5g2-F IRF5g2-R	AGGACTGCACTGGCTCAATCAAGA ATCTACTCCTTCCTGATACTTGCCT	For the second intron	
IRF5g3-F IRF5g3-R	ATGGAAAGCCAATCTCCGCTGTG GTAACATGAGCGGACTGCTCAGC	For the third, fourth and fifth intron	
IRF5g4-F IRF5g4-R	AGTGCAATCAAAGTTGAGCAGGCA ATGATCGGTATAGAACCTCTGCTT	For the sixth intron	
IRF5g5-F IRF5g5-R	ACGCCATCAGGCTGTGCCAGTGTA CAATGATGAGCTTTTTCTCCTTG	For the seventh intron	
IRF5g6-F IRF5g6-R	GCTTCGGTGAGGACTGGCCAGACA ACTCTGCAGAAGTCTGTGGAGCT	For the eighth intron	
IRF5-rF IRF5-rR	CCTGGGCTCAGAACATCCACTAAC AGTATGGCATCATAGAGGGCACCT	Real-time PCR	
TNFα-F TNFα-R	ACAGGTGATGGTGTCGAGGAGGA TCTGAGACTTGTTGAGCGTGAAG	Real-time PCR	JF957372
S11-F S11-R	CCGTGGGTGACATCGTTACA TCAGGACATTGAACCTCACTGTCT	Real-time PCR	AB012087
ISG15-F	GTGAGCGGTGAAGCCACAGTTG	Real-time PCR	KP115358
ISG15-R	GCGAACCGTTATCGGCAGACAG		
PKR-F	AGGCTTGATCCACAGAGACCTGAA	Real-time PCR	JX516101
PKR-R	CGTTCCAGAAGTTGCACGTCATTG		

PCR products were analysed by electrophoresis on a 1 % agarose gel and the anticipated fragments were purified from agarose gels. These fragments were ligated into the pMD18-T vector (TaKaRa) and transformed into competent *Escherichia coli* DH-5α competent cells, and recombinants were identified and sequenced (Invitrogen).

### Sequence and phylogenetic analysis

The full-length cDNA sequence of ccIRF5 was compared with other corresponding IRF5 sequences by using the BLAST program from the National Center for Biotechnology Information (NCBI) [24] and the MegAlign program DNASTAR. The putative ORF and the protein prediction were analysed with Editseq within DNASTAR. The protein domains were predicted with simple modular architecture research tool (SMART) [25]. Phylogenetic analysis was performed with MEGA 5.0, using the neighbor-joining method. Homologous sequences were searched using the NCBI BLAST server.

### Leukocyte isolation and in vitro challenges

The methods for isolating common carp leukocytes from peripheral blood (PBLs) and head kidney (HKLs) have been described by Rymuszka et al. [26]. Peripheral blood was diluted 1:1 with Leibovitz’s L-15 medium (supplemented with 10 U/ml of heparin, 10 mM HEPES, 60 mM NaCl, 5 % FBS, 100 U/ml penicillin, 100 μg/ml streptomycin, and 250 ng/ml amphotericin B) and layered on a 65 % Percoll (Sigma) solution, and centrifuged at 2500 rpm for 30 min at 4 °C. The cells were washed by centrifugation in PBS and re-suspended in cold Leibovitz’s L-15 medium.

Head kidney cell suspensions were obtained by gently pressing the tissue with a plunger through a 100-μm sterile nylon mesh with Leibovitz’s L-15 medium. Percoll layers of 51 and 34 % were used and centrifuged at 1500 rpm for 30 min at 4 °C. The cell layers at the interphase were collected and washed twice with Leibovitz’s L-15 medium.

For stimulation experiments, 1 × 10^6^ cells were maintained at 25 °C in a 24-well tissue culture plate with 0.5 mg/ml poly I:C and 1 mg/ml LPS (suspended in PBS), while un-challenged cells served as controls (indicated as 0 h). The cells were harvested at 0, 3, 6, 9, 12 and 24 hpi and RNA was extracted and reverse transcribed.

### Real-time quantitative PCR

The inducible expression profiles of ccIRF5, ccTNFα and two ISG genes [ccISG15 and ccPKR (protein kinase R)] upon poly I:C and LPS challenges were analysed by real-time PCR using gene-specific primers (Table 1) on an iQ5 Real-time PCR instrument (Bio-Rad). Reactions were performed in a 20 μl volume using SYBR Green RealMasterMix (TIANGEN). Cycling conditions were one cycle of 94 °C for 5 min, followed by 40 cycles of 94 °C for 20 s, 60 °C for 30 s and 72 °C for 20 s. The 40S ribosomal protein S11 gene was amplified in parallel as a housekeeping control for normalization [27]. The amplification efficiency of the primers used in the real-time PCR was between 0.80 and 0.86. All samples were analysed in triplicate. Standard curves were run on the same plate. The real-time PCR data were analysed with the 2^-△△CT^ method.

### Statistical analysis

Differences in relative gene expression between the challenged group and the control group were tested using one-way analysis of variance (ANOVA) in Graphpad Prism 5. *P* values < 0.05 were considered statistically significant.

## Results

### Characterization of the cDNA sequence of ccIRF5

**Fig. 1:**
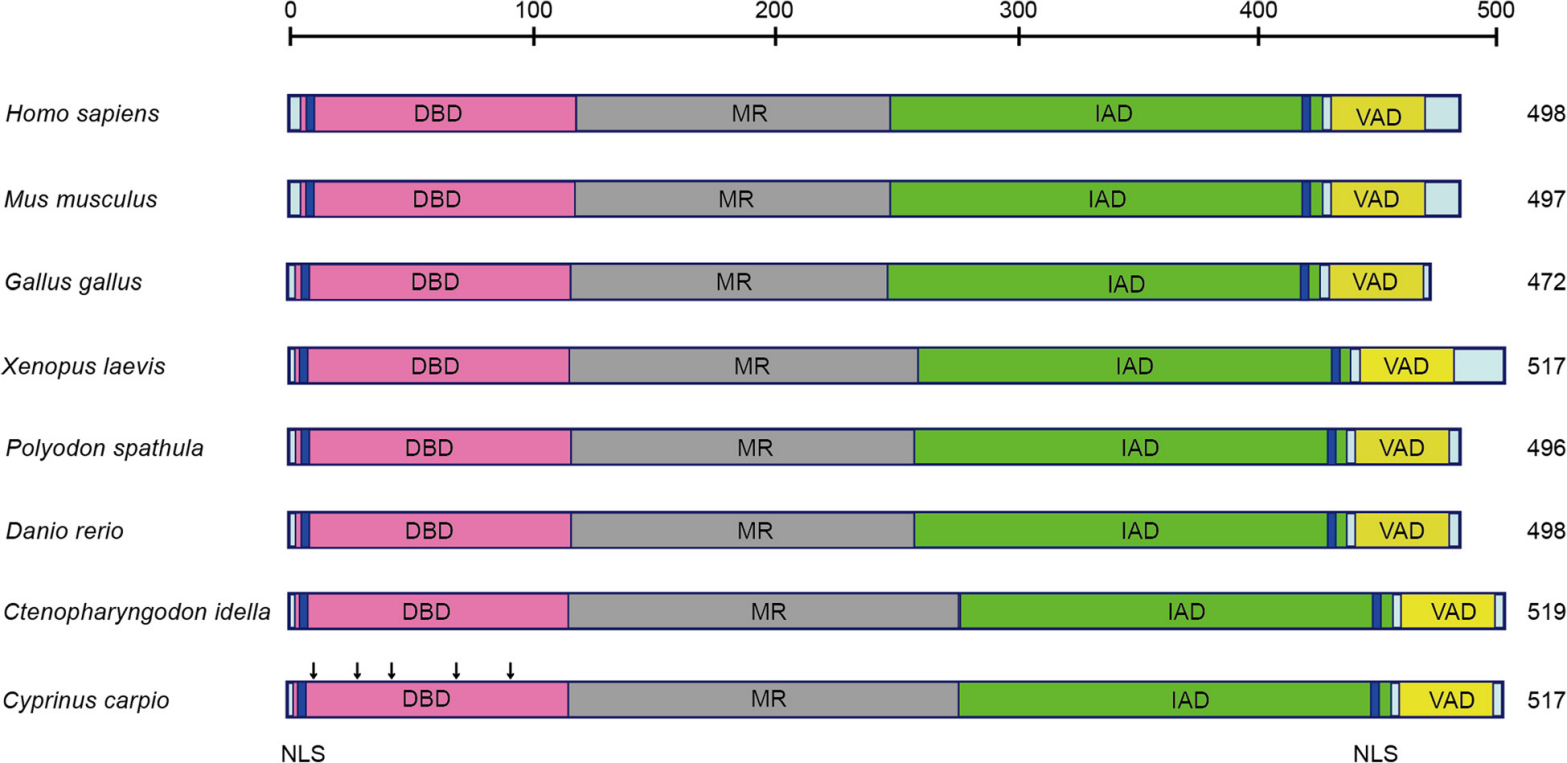
Domain organization of ccIRF5 and other vertebrate IRF5 proteins. The DBD (*pink*), MR (*grey*), IAD (*green*) and VAD (*yellow*) are depicted in different colours. The two NLSs are boxed in blue and the conserved tryptophan residues are indicated by downward arrowheads. Numbers refer to the length of the amino acid sequences. The accession numbers are listed in Table 2

The full-length cDNA of ccIRF5 (GenBank Accession No.: KP979609) was 2042 bp, consisting of a 5’-untranslated region (UTR) of 266 bp, a 3’-UTR of 222 bp with a polyadenylation signal (AATAAA) starting 30 bp upstream of the poly (A) tail and an ORF of 1554 bp encoding a peptide of 517 amino acids. The predicted molecular weight of ccIRF5 is 58.5 kDa, and the isoelectric point is 5.8. The deduced amino acid sequence of ccIRF5 shares higher identities with the fish IRF5s (65.4 % of *P. spathula* to 90.0 % of *C. idella*) than with IRF5s of other species (51.6 % of *G. gallus* to 56.7 % of *M. musculus*) (Table 2) and contains a DBD, a middle region (MR), an IAD and a virus activated domain (VAD) (Fig. 1).

**Table 2:** Amino acid identities (%) of ccIRF5 to other vertebrate IRF5 proteins

Species	Protein length (aa)	GeneBank No.	Full-length identities	DBD identities
*Ctenopharyngodon idella*	519	ACT83675	90.0	96.5
*Danio rerio*	498	ABY91289	87.9	93.9
*Ictalurus Punctatus*	478	AHH37262	76.8	85.1
*Paralichthys olivaceus*	472	AEY55357	68.9	75.4
*Scophthalmus maximus*	487	AEG76960	69.7	75.4
*Salmo salar*	532	NP_001133324	69.6	82.5
*Oplegnathus fasciatus*	498	AFZ93894	69.7	80.7
*Miichthys miiuy*	492	AHB59743	70.1	80.7
*Polyodon spathula*	496	AEW27153	65.4	74.6
*Xenopus laevis*	517	NP_001088065	56.9	73.7
*Gallus gallus*	472	NP_001026758	51.6	58.8
*Mus musculus*	497	AAB81997	56.7	67.5
*Homo sapiens*	498	AAH04201	55.3	67.5

### Genomic structure of ccIRF5

**Fig. 2:**
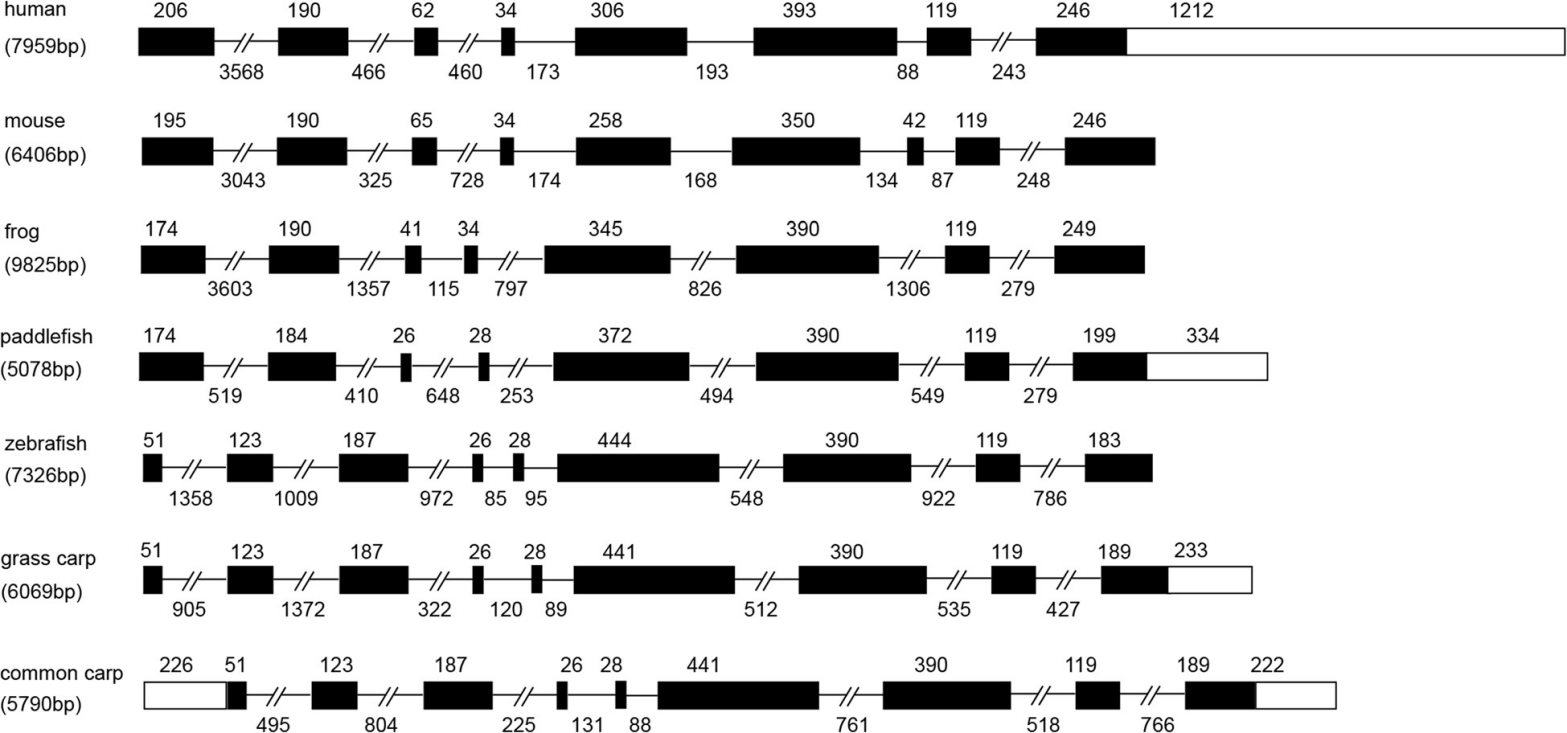
Schematic diagram of exon-intron arrangement of IRF5 genes from various vertebrates. Exons are indicated by boxes and introns by straight or interrupted lines (>200 bp). The numbers above the boxes and below the lines indicate the lengths of exons and introns, respectively. Sequences selected are the following IRF5s: Human (Gene ID 3663), Mouse (Gene ID 27056), Paddlefish (Gene ID JF511655), Zebrafish (Gene ID 405811), Grass carp (GenBank: FJ556995) and Common carp (GenBank: KP979609)

The ccIRF5 gene is 5798 bp and is composed of 9 exons and 8 introns (Fig. 2). The exon-intron splice junctions follow the AG/GT rule (Table 3). The ccIRF5 gene has the same size exons as the grass carp IRF5, while, the introns sizes are different, which are 495, 804, 225, 131, 88, 761, 518, and 766 bp (listed in Additional file 1).

**Table 3:** Intron-exon junctions and flanking sequences of the ccIRF5 gene

Exon no.	Position in DNA	Exon size (bp)	Splice donor	Splice acceptor	Intron size (bp)
1	1–277	277	**gt**gaattgtg	accctgcc**ag**	495
2	773–896	123	**gt**gagtaata	tgctcttc**ag**	804
3	1701–1888	187	**gt**aaggaccg	ttgatttt**ag**	225
4	2114–2140	26	**gt**aagttctg	tgcctttc**ag**	131
5	2272–2300	28	**gt**aagatttt	tgcttgac**ag**	88
6	2389–2830	441	**gt**aagcctct	tcttctct**ag**	761
7	3592–3982	390	**gt**aagaggaa	ttcttctc**ag**	518
8	4501–4620	119	**gt**ttggagat	cgttatct**ag**	766
9	5487–5798	411			

Bold text indicates the invariant nucleotides of the exon-intron boundaries

### Phylogenetic analysis

**Fig. 3:**
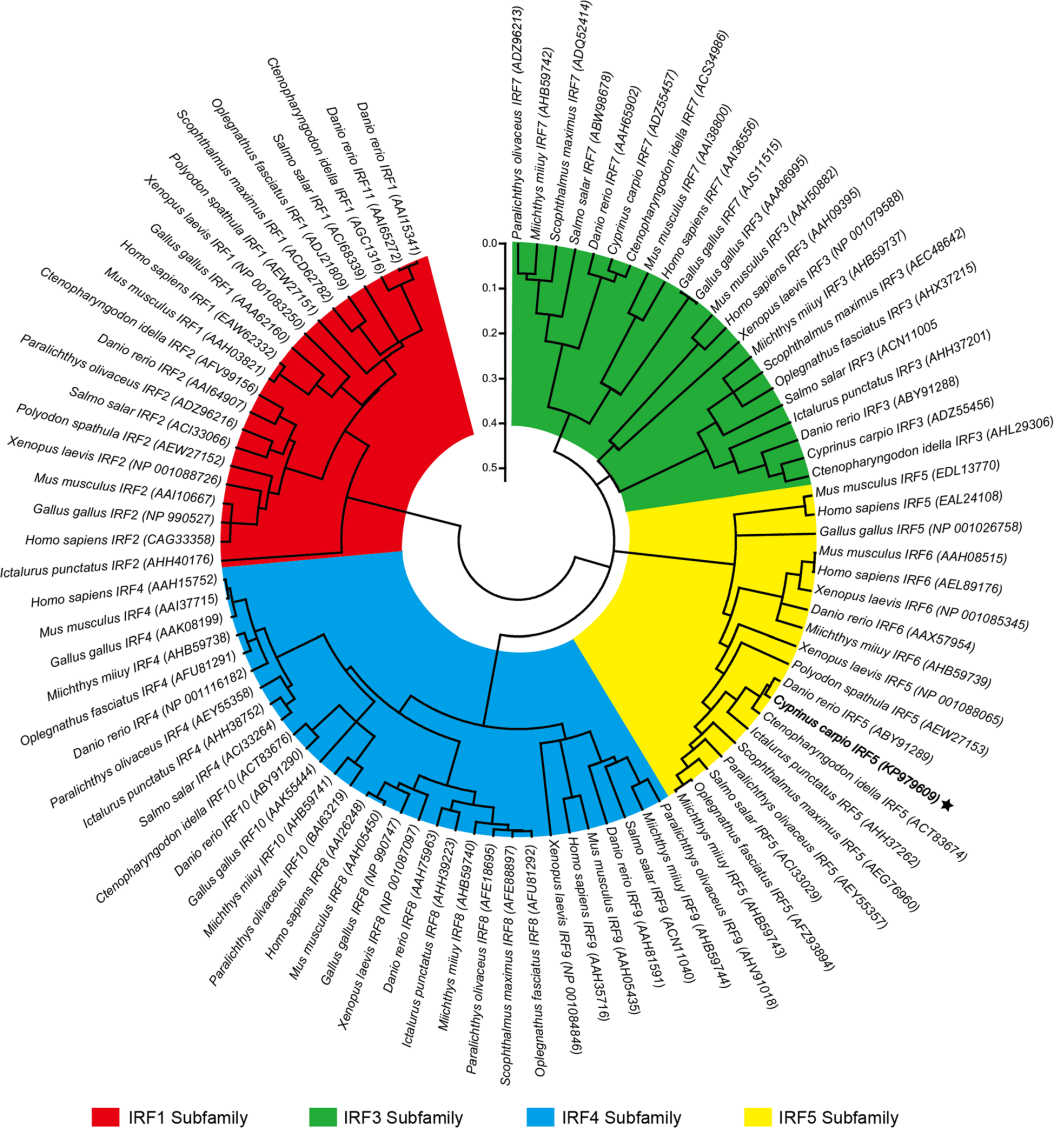
Phylogenetic tree of ccIRF5 with known IRFs in vertebrates. The tree was constructed using the neighbor-joining method within the MEGA 5.0. Numbers above the nodes are bootstrap values on 1000 replicates. The GenBank accession numbers for the IRF sequences are shown within the brackets next to each species

The phylogenetic tree was constructed based on a Clustal W alignment using MEGA 5.0 by the neighbor-joining method. All IRF members were divided into four subfamilies: IRF1, IRF2, and zebrafish IRF11 belonged to the IRF1 subfamily, IRF3 and IRF7 belonging to the IRF3 subfamily; IRF4, 8, 9, and 10 belonging to the IRF4 subfamily; IRF5 and IRF6 belonging to the IRF5 subfamily. In the phylogenetic tree, ccIRF5 showed a closer relationship with genes from other cyprinid fish, including grass carp and zebrafish IRF5s, and clustered into the fish IRF5 subgroup (Fig. 3).

### Tissue distribution of ccIRF5 mRNA in healthy carps

**Fig. 4:**
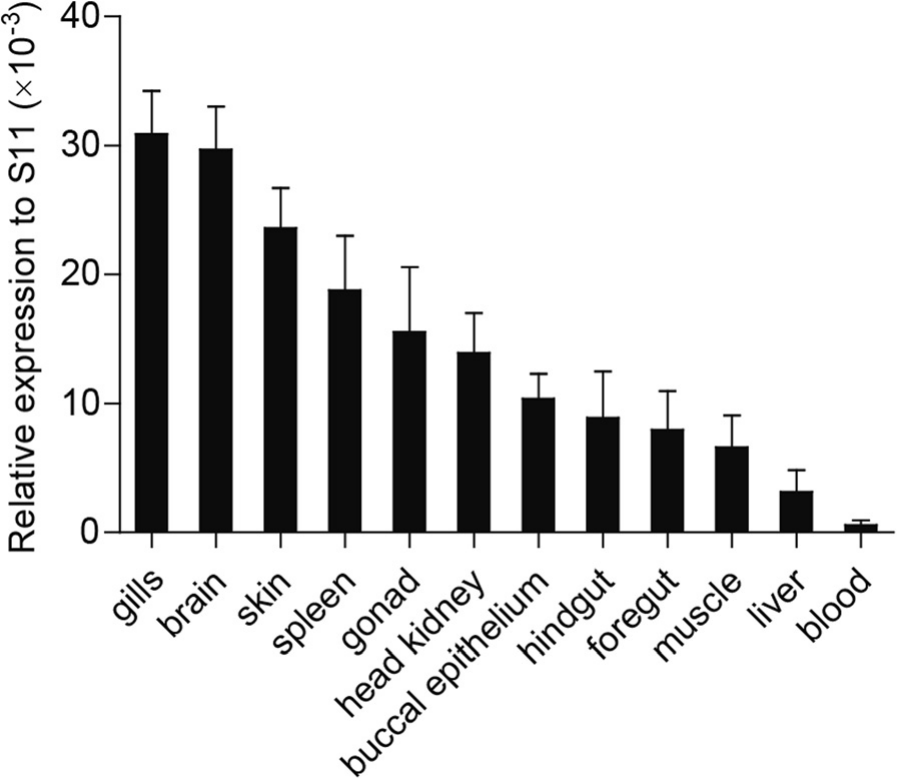
Tissue distribution analysis of ccIRF5 mRNA by real-time PCR. Total RNA was isolated from various tissues of healthy carps. Gene expression levels were normalized to 40S ribosomal protein S11 mRNA. The data are presented as the mean ± S.D. (*n* = 3)

Constitutive expression of ccIRF5 mRNA in various tissues (gills, brain, skin, spleen, gonad, head kidney, buccal epithelium, hindgut, foregut, muscle, liver and blood) of healthy carps was examined by real-time PCR. The highest expression levels were in the gills and the brain, followed by the skin, the spleen, the gonad, the head kidney, the buccal epithelium, the hindgut, the foregut and the muscle, while low expression was observed in the liver and the blood (Fig. 4).

### Gene expression of ccIRF5 in response to poly I:C challenge in vivo

**Fig. 5:**
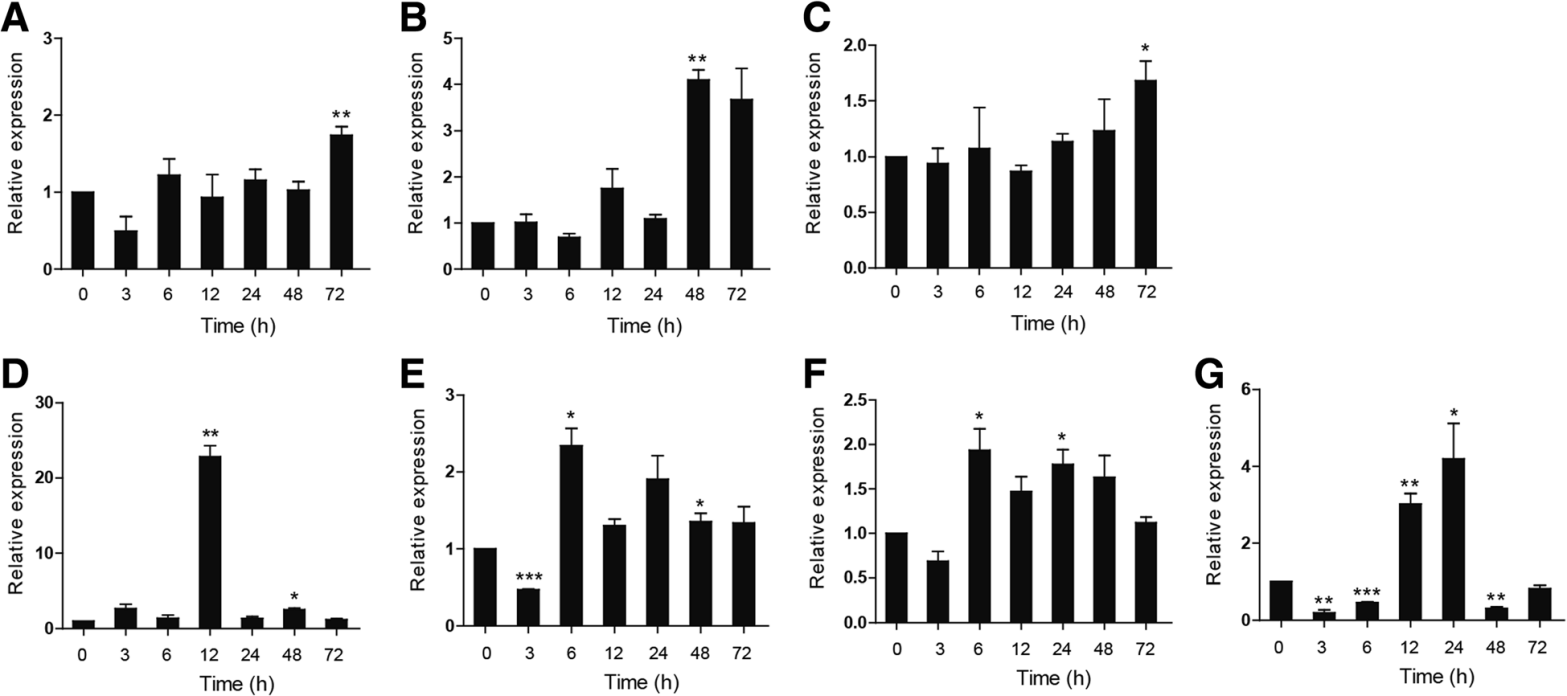
Expression analysis of ccIRF5 in response to poly I:C challenge in vitro. Total RNA was extracted from head kidney (**a**), liver (**b**), spleen (**c**), gill (**d**), foregut (**e**), hindgut (**f**) and skin (**g**) of the control and challenged samples at 0, 3, 6, 12, 24, 48 and 72 hpi. Expression was normalized to S11 and shown as relative to control. The data are presented as the mean ± S.D. (*n* = 3). Significant values in comparison to the control are indicated by *p < 0.05, **p < 0.01 and ***p < 0.001

Temporal expression of ccIRF5 upon poly I:C challenge in seven immune-related tissues (liver, spleen, head kidney, foregut, hindgut, skin and gills) was examined at 0, 3, 6, 12, 24, 48 and 72 hpi using real-time PCR. The highest expression levels in different tissues were reached at different time points. The maximum induction of ccIRF5 in mucosa-associated tissues including the foregut, the hindgut, the skin and the gills, occurred within 24 hpi. In contrast, the expression levels in the spleen, the head kidney and the liver were up-regulated at later time points (48 and 72 hpi). The expression of ccIRF5 was weakly up-regulated in all tested tissues (1.7- to 4.2-fold, *p* < 0.01 or *p* < 0.05) except in the foregut (22.8-fold increase, *p* < 0.01) (Fig. 5).

### Gene expression of ccTNFα and ccISGs in response to poly I:C challenge in vivo

**Fig. 6:**
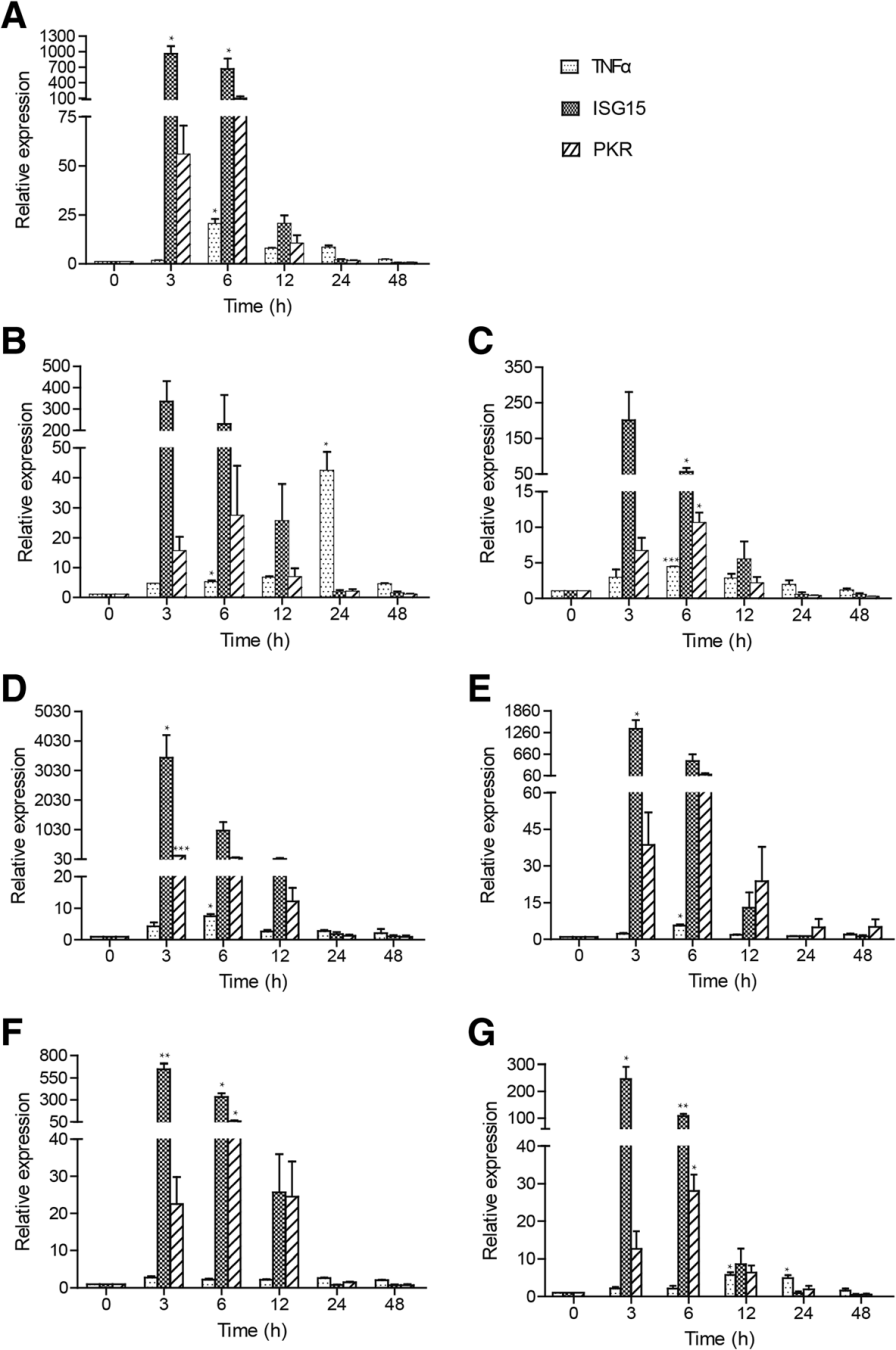
Expression analysis of ccTNFα and ccISGs in response to poly I:C challenge *in vitro*. Total RNA was extracted from head kidney (**a**), liver (**b**), spleen (**c**), gill (**d**), foregut (**e**), hindgut (**f**) and skin (**g**) of the control and challenged samples (shown in Fig. 5). The expression was normalized to S11 and shown as relative to control. The data are presented as the mean ± S.D. (*n* = 3). Significant values in comparison to the control are indicated by *p < 0.05, **p < 0.01 and ***p < 0.001

The expression levels of ccTNFα, ccISG15 and ccPKR in all tested tissues were increased at different time points upon stimulation with poly I:C. Subsequently, the expression levels decreased gradually until 48 hpi. The highest induction of ccTNFα was detected in the spleen (42.3- fold, *p* < 0.01) and liver (20.4-fold, *p* < 0.01), respectively. Whereas, the expression levels of ccTNFα in the skin (2.8-fold) and the gills (5.8-fold, *p* < 0.05) were weakly up-regulated. The expression of ccISG15 and ccPKR in all tested tissues reached peak levels at 3 and 6 hpi, respectively. Fold induction of ccISG15 was significantly increased in the foregut (3464.8-fold, *p* < 0.05) and hindgut (1364.1-fold, *p* < 0.05). Furthermore, the highest expression of ccPKR was observed in the liver and the hindgut at 6 hpi by 95.9-fold and 88.1-fold, respectively (Fig. 6).

### Expression profiles of ccIRF5 upon poly I:C and LPS challenges in vitro

**Fig. 7:**
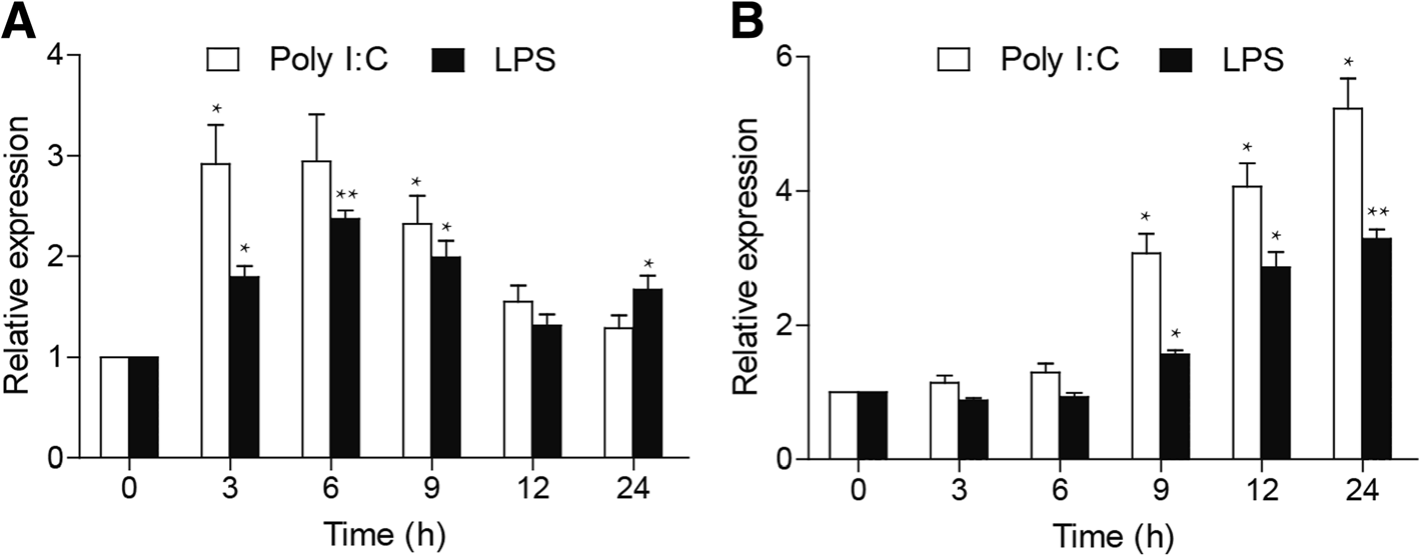
Expression levels of ccIRF5 in the PBLs (**a**) and HKLs (**b**) induced by ploy I:C and LPS in vivo. Total RNA was extracted from control and challenged samples at 0, 3, 6, 9, 12 and 24 hpi. The expression was normalized to S11 and shown relative to control. The data are presented as the mean ± S.D. (*n* = 3). Significant values in comparison to the control are indicated by *p < 0.05 and * *p < 0.01

To further investigate the antiviral and antibacterial response of ccIRF5 in vitro, common carp PBLs and HKLs were isolated and challenged them with poly I:C or LPS. In accordance with the in vivo studies, ccIRF5 transcripts in the common carp PBLs reached the peak level at 3 hpi (2.9-fold, *p* < 0.05) and 6 hpi (2.0-fold *p* < 0.05), upon poly I:C and LPS challenges, respectively. As well as in the HKLs, expression of ccIRF5 reached the peak level at 24 hpi by 5.2-fold and 3.3-fold upon poly I:C and LPS challenges, respectively (Fig. 7).

### Expression profiles of ccTNFα and ccISGs in poly I:C- and LPS- stimulated PBLs

**Fig. 8:**
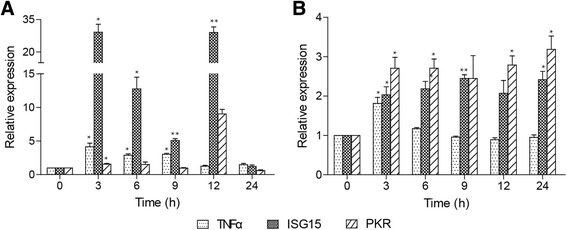
Expression analysis of ccTNFα and ccISGs in common carp PBLs upon poly I:C (**a**) and LPS (**b**) challenges. Total RNA was extracted from the challenged samples at each time point (shown in Fig. 7). The expression was normalized to S11 and shown relative to control. The data are presented as the mean ± S.D. (*n* = 3). Significant values in comparison to the control are indicated by *p < 0.05 and **p < 0.01

All three genes were up-regulated at 3 hpi by both poly I:C and LPS challenges in the PBLs. However, compared to the challenge by poly I:C (4.1- to 29.1-fold), expression levels of the three genes were weakly induced upon LPS challenge (1.8- to 3.2-fold) (Fig. 8).

## Discussion

The IRF family of transcription factors plays an important role in the regulation of type I IFN genes and ISGs. IRF5 genes have been identified in several vertebrates. However, there is no evidence about the identification and function of IRF5 in common carp. In the present study, we cloned the full-length cDNA and genomic sequence of ccIRF5. The deduced amino acid sequence contains a DBD, a MR, an IAD and a VAD (Fig. 1). Similar to other IRF5s in vertebrates, five tryptophan residues (W13, W28, W40, W60 and W79) are present in the DBD which forms a helix-turn-helix structure to bind to the IFN stimulated response element (IRSE)/IRF binding element (IRF-E) consensus in the target promoters [28]. The VAD that comprises all serine residues function as virus-induced phosphorylation sites. The MR which contains a proline-rich domain and is less conserved. Although this domain is also present in IRF3, 4 and 6, its function is still unclear [29]. The IAD, which was originally identified in IRF8, can form transcriptional complexes with other IRFs or transcriptional co-modulators to initiate the transcription of target genes [7]. Chen W et al. reported that the dimerization between hydrophobic and ionic interactions in the IAD played a crucial role in the high basal activity of IRFs [30]. Similar to other IRF5s, two nuclear localization signals (NLSs) are found in the N- and C-termini of the predicted ccIRF5 protein, and these NLRs play an important role in IRF nuclear translocation and retention in virus-infected cells [11, 14, 17].

Similar to IRF5 genes of other Cypriniformes (zebrafish and grass carp), the genomic sequence of ccIRF5 is also composed of 9 exons and 8 introns. However, the IRF5 genes in human and several other vertebrates contain 8 exons and 7 introns. Interestingly, the sizes of the first and second exons, which encode the DBD of IRF5s in common carp, zebrafish and grass carp, are similar to the size of the first exon in other vertebrates, while the other exons sizes comparable to exons in vertebrates (Fig. 2). These results suggest that the genomic structure of vertebrate IRF5s is evolutionarily conserved, and the first intron of IRF5s in common carp, zebrafish and grass carp may be lost in some other teleosts and tetrapods during evolution.

The phylogenetic tree showed that all IRF family members were divided into four subfamilies, and ccIRF5 showed a closer relationship with other cyprinids, including grass carp and zebrafish IRF5s, which were well clustered into the fish IRF5 subgroup (Fig. 3). This result matches the evolutionary relationship observed at the genomic structure level for the IRF5s in all species (Fig. 2).

The ccIRF5 transcripts were ubiquitously expressed in all tested tissues of healthy carp, which was similar to the expression of other known fish IRFs [14, 31–35]. The highest ccIRF5 expression levels in healthy common carp were in the gills and the brain, while low expression was observed in the liver and the blood (Fig. 4). Similarly, the highest expression levels of Japanese flounder and paddlefish IRF5 were also detected in the gills, which are mucosa-associated lymphoid tissues that harbour lymphocytes [17, 21]. These results suggest that IRF5 may be crucial role for the activity of the mucosal immune system in fish. Turbot IRF5 was highly expressed in the brain, with which our result is in more agreement, suggesting that IRF5 might also play a significant role in the central nervous system [36]. High expression levels of IRF5 were shown in various tissues of different fish species. For instance, grass carp and half-smooth tongue sole IRF5s were expressed in all examined organs and the highest expression was in the spleen [14, 19]. The rock bream IRF5 gene was highly expressed in the liver [18]. In contrast, the highest expression levels of IRF5 in zebrafish were in the ovaries and the muscle, which are not immune-associated tissues [15]. The reason for the dissimilarities in IRF5 expression patterns in different fish species may be due to the diverse immune systems of fishes.

Previous studies have highlighted the important role of IRF5 in regulating the expression of IFN and other pro-inflammatory cytokines in TLR7 and 8 antiviral responses [13]. To gain insight into the role of ccIRF5 in response to the treatment with poly I:C, which is a synthetic double-stranded RNA, the temporal expression of ccIRF5 in seven immune-related tissues was examined. As shown in Fig. 5, the expression levels of ccIRF5 in different tissues reached peak levels at different time points. The maximum induction of ccIRF5 in mucosa-associated tissues including the foregut, the hindgut, the skin and the gills, occurred within 24 hpi compared to the expression levels in the spleen, the head kidney and the liver (at 48 and 72 hpi). This phenomenon may be because the fish mucosal immune system is the first line of defence against the invading pathogens. The expression of ccIRF5 was slightly up-regulated in all tested tissues (1.7- to 4.2-fold), with the exception of the foregut (22.8-fold). Similarly, the expression levels of IRF3 and IRF7 mRNA were highly up-regulated in the intestine of spring viraemia of carp virus (SVCV)-infected common carps [22]. These results may indicate that the IRF family plays a key role in the intestinal immune system. The expression levels of IRF5 in the immune and non-immune tissues of zebrafish, rock bream and turbot were weakly affected by infectious poly I:C, which is in accordance with our study [15, 18, 36]. In contrast, the induction magnitude of IRF5 was much stronger in the tissues of poly I:C-injected Japanese flounder [17]. In addition, fish (grass carp, Japanese flounder, rock bream, turbot and half-smooth tongue soles) IRF5 genes were reported to be significantly induced by different pathogens (grass carp reovirus, lymphocystis disease virus, iridovirus, turbot reddish body iridovirus and megalocytivirus) [14, 15, 17–19, 36]. These results indicate that fish IRF5 may respond to different pathogens in a tissue- or virus-specific manner, but the mechanism involved require further investigation.

TNFα, a potent proinflammatory cytokine produced following PAMP recognition by PRRs, and ISGs were also observed at different time points upon the stimulation of poly I:C in vivo by real-time PCR. The highest induction of ccTNFα was detected in the spleen and the liver at 24 hpi (42.3- fold) and 6 hpi (20.4-fold), respectively (Fig. 6). This finding is potentially because the spleen and liver are key innate immune tissues in fish that have a rich resident population of macrophages and lymphocytes, which secrete large quantity of TNFα upon stimulation with pathogens. The Skin, the gills and the intestine (including foregut and hindgut) are important mucosal lymphoid tissues in fish [37]. In this study, following poly I:C challenge, ccISG15 and ccPKR in the foregut and the hindgut were significantly induced. This result indicates that IRF5 might play an important role in mucosal immune system. Further, in our preliminary studies, gene expression of ccIFN and ccMx in all tested tissues was also significantly induced by poly I:C stimulation (unpublished data). In Japanese flounder and turbot, Mx expression was strongly induced by poly I:C in the head kidney and the gills [38, 39]. Thus, these results suggest a role of ccIRF5 in the activation of downstream antiviral pathways.

In accordance with the in vivo study, ccIRF5 transcripts in common carp PBLs and HKLs were induced upon the stimulation of poly I:C and LPS (Fig. 7). The reason for this phenomenon might because of the presence of immune cells, including monocytes, granulocytes and lymphocytes [40, 41]. Notably, the highest induction of ccIRF5 in the PBLs (3 to 6 hpi) was earlier than the induction in the HKLs (24 hpi). This might be due to that IRF family members perform different cellular activities in a cell type-specific way [42].

Mx and TNFα were induced upon transfection of IRF5 in rock bream heart cells [18]. In this study, ccTNFα, ccISG15 and ccPKR transcripts in common carp PBLs were significantly up-regulated upon the two stimuli (Fig. 8). Intriguingly, following LPS stimulation, ccISG15 and ccPKR expression reached the highest level at 24 hpi, which was later than the stimulation by poly I:C. And the fold change of the three genes induced by poly I:C (4.1- to 29.1-fold) was stronger than that induced by LPS (1.8- to 3.2-fold). This phenomenon may be due to that IRF5 mediates immune response in a pathogen-specific way. In accordance with our study, expression of IRF5 in half-smooth tongue sole was significantly increased post bacterial infection in vivo [19]. Therefore, these results are signifying ccIRF5 may be not only play an the important role in regulating the antiviral immune response, as reported for mammalian IRF5, but also can respond to bacterial challenge [12, 43].

## Conclusions

In summary, we report the cloning and characterization of an IRF5 gene from common carp at transcriptional and genomic DNA levels. Furthermore, we describe tissue distribution and in vivo and in vitro induction of ccIRF5, ccTNFα and ccISGs upon stimulation with poly I:C and LPS. Our findings suggest that IRF5 might play an important role in regulating the antiviral and antibacterial response in fish, and these results could provide a clue for preventing common carp infection by pathogenic microorganisms present in the aquatic environment.

## Abbreviations

ANOVA, analysis of variance; cc, common carp; DBD, DNA binding domain; HKLs, leukocytes from head kidney; hpi, hours post-injection; IAD, interferon regulatory factor-associated domain; ICSAT, interferon consensus sequence-binding protein in adult T-cell leukemia cell line or activated T cells; ICSBP, interferon consensus sequence binding protein; IFN, interferon; IL, interleukin; IRF, interferon regulatory factor; IRF-E, interferon regulatory factor binding element; IRSE, interferon stimulated response element; ISG, interferon stimulated gene; ISGF3γ, interferon stimulated gene factor 3 gamma; LPS, lipopolysaccharides; LSIRF, lymphoid-specific interferon regulatory factor; MR, middle region; NCBI, National Center for Biotechnology Information; NLSs, nuclear localization signals; ORF, open reading frame; PBLs, leukocytes from peripheral blood; PBS, phosphate-buffered saline; PIP, PU.1 interaction partner; PKR, protein kinase R; poly I:C, polyinosinic:polycytidylic acid; RACE, rapid amplification of the cDNA ends; RIG-I, retinoic acid-inducible gene I; SMART, simple modular architecture research tool; SVCV, spring viraemia of carp virus; TNFα, tumour-necrosis factor α; UTR, untranslated region; VAD, virus activated domain.

## Additional file

Additional file 1: Intron sequences of the ccIRF5 gene. Intron sizes are 495, 804, 225, 131, 88, 761, 518, and 766 bp. (TXT 3 kb)
